# Rosmarinic Acid Improves Cognitive Abilities and Glucose Metabolism in Aged C57Bl/6N Mice While Disrupting Lipid Profile in Young Adults in a Sex-Dependent Fashion

**DOI:** 10.3390/nu15153366

**Published:** 2023-07-28

**Authors:** Chiara Musillo, Letizia Giona, Michael Ristow, Kim Zarse, Karsten Siems, Alessia Di Francesco, Barbara Collacchi, Carla Raggi, Francesca Cirulli, Alessandra Berry

**Affiliations:** 1 Center for Behavioural Sciences and Mental Health, Istituto Superiore di Sanità, Viale Regina Elena 299, 00161 Rome, Italy; chiara.musillo@iss.it (C.M.); letizia.giona@guest.iss.it (L.G.); difrancescoalessia@libero.it (A.D.F.); barbara.collacchi@iss.it (B.C.); 2 Institute of Experimental Endocrinology and Diabetology, Charité Universitätsmedizin Berlin, 10117 Berlin, Germany; michael.ristow@charite.de (M.R.); kim.zarse@charite.de (K.Z.); 3 AnalytiCon Discovery GmbH, 14473 Potsdam, Germany; k.siems@ac-discovery.com; 4 National Center for the Control and Evaluation of Medicine, Istituto Superiore di Sanità, 00161 Rome, Italy; carla.raggi@iss.it

**Keywords:** rosmarinic acid, aging, mouse model, oxidative stress, sex-differences, cognitive abilities, metabolism

## Abstract

A growing body of evidence suggests that regular consumption of natural products might promote healthy aging; however, their mechanisms of action are still unclear. Rosmarinic acid (RA) is a polyphenol holding anti-inflammatory, antioxidant and neuroprotective properties. The aim of this study was to characterise the efficacy of an oral administration of RA in promoting healthspan in a mouse model of physiological aging. Aged C57Bl/6 male and female (24-month-old) mice were either administered with RA (500 mg/Kg) or a vehicle in drinking bottles for 52 days while 3-month-old mice receiving the same treatment were used as controls. All subjects were assessed for cognitive abilities in the Morris water maze (MWM) and for emotionality in the elevated-plus maze test (EPM). Brain-derived Neurotrophic Factor (BDNF) protein levels were evaluated in the hippocampus. Since the interaction between metabolic signals and cerebral functions plays a pivotal role in the etiopathogenesis of cognitive decline, the glycaemic and lipid profiles of the mice were also assessed. RA enhanced learning and memory in 24-month-old mice, an effect that was associated to improved glucose homeostasis. By contrast, the lipid profile was disrupted in young adults. This effect was associated with worse glycaemic control in males and with reduced BDNF levels in females, suggesting powerful sex-dependent effects and raising a note of caution for RA administration in young healthy adult subjects.

## 1. Introduction

Aging is characterised by a progressive loss in the ability to maintain homeostasis, becoming itself a main risk factor for health outcomes in later life. While *gerontoscience* was traditionally oriented towards interventions aimed at prolonging “lifespan”, the current challenge is to identify suitable strategies to promote “healthspan”—namely, the number of years spent in good health during aging [1,2,3,4]. In this specific context, we have recently provided a framework to define the elusive concept of health (and thus of healthspan) by considering a number of functional domains that are widely shared between the most popular preclinical models used to study the aging process and humans. These domains include, among the others, stress resistance and homeostasis (summarized as physiological function), in addition to cognition (cognitive function), features that can be targeted by intervention strategies aimed at buffering oxidative stress [1,4].

Indeed, a growing body of evidence suggests that the regular consumption of natural products (vegetables, fruits, leaves, roots, seeds, berries, etc.) might promote healthy aging by activating signalling pathways, triggering adaptive stress responses and resulting in anti-inflammatory, anti-oxidant and in general geroprotective effects [5,6]. For this reason, nutraceuticals have attracted the interest of the scientific community, with the main aim of alleviating the burden of age-related pathologies [7]. Rosmarinic acid (RA) is a polyphenol found in several species of the Lamiaceae family holding anti-inflammatory, antioxidant and neuroprotective properties (see [8] and references therein). Lamiaceae are broadly used as cooking herbs and as medicinal plants in many ethnopharmacological traditions together with oregano, sage, rosemary, thyme and lavender showing the greater diffusion in the Mediterranean area [9]. RA can increase lifespan in C. elegans [10]; very recent in vitro evidence suggests that RA holds antidiabetic and anticholinergic properties [11], while in vivo studies provide evidence for RA to improve cognitive functions in a mouse model of Alzheimer Disease (AD) [12] and to be able to modulate cholesterol in a murine model of high-fat-diet-induced obesity [13]. Thus, RA appears to be a promising compound with overall appealing pro-healthspan features, although a more thorough characterisation is still needed.

The aim of this study was to characterise the efficacy of an oral administration of RA in promoting healthspan in a mouse model of physiological aging (i.e., characterised by the absence of overt pathologies) compared to healthy adult mice. Since cognitive decline is a highly disabling and prevalent condition in the aging population, greatly affecting physical health and quality of life, we primarily focused on the ability of RA to counteract aging-related cognitive decline on 24-month-old mice by means of the Morris water maze (MWM) test. Changes in the emotional profile were also assessed in the elevate plus-maze (EPM) to dissect the contribution of possible aging-related anxiety-like behaviours on learning and memory performances. A great body of evidence suggests that the interaction between metabolic signals and cerebral functions plays a pivotal role in the etiopathogenesis of cognitive decline [14,15,16]. Thus, the metabolic phenotype was thoroughly characterised by evaluating both the glucose and lipid profiles. More in detail, changes in glucose homeostasis were assessed through the glucose tolerance and insulin sensitivity tests, while fructosamine and glucose levels as well as triglycerides, free fatty acids and cholesterol, were measured in peripheral blood upon sacrifice. Moreover, BDNF, a neurotrophin involved in brain plasticity, neurogenesis, memory consolidation as well as in the control of metabolism, was also assessed in the hippocampus, a brain region playing a main role in cognitive abilities [17].

It has been hypothesised that a mortality–morbidity-paradox in humans exists such that despite living longer than men (on average), women appear overall to be in poorer health and reduced healthspan. Such a hypothesis might also hold true in species characterised by a similar sex bias in mortality as mice [18]. Thus, to evaluate more in detail potential sex-dependent effects, the efficacy of RA was tested both in male and female mice.

## 2. Materials and Methods

This study was reported in conformity with ARRIVE guidelines [19].

### 2.1. Experimental Subjects

Aged 18-month-old (32 males and 33 females) and young 3-month-old (21 males and 23 females) C57Bl6/N mice were purchased from Charles River. In this experiment, young mice were used as a control for the effect of RA to counteract the age-related changes in the behavioural and metabolic phenotype. Aged subjects were left undisturbed in the animal facility until they reaching 24 months of age. When reaching 22 months of age, 14 males and 15 females were used in a pilot study aimed at assessing RA solubility in water and its palatability; in addition, possible toxic effects of the selected dose were assessed by measuring alanine aminotransferase—ALAT—in liver (see Supplementary Materials). When reaching 24 months of age, 6 males and 3 females died while 12 males and 15 females became experimental subjects. Animals were group-housed (3/4 per cage) with same sex and age conspecifics in transparent Plexiglas cages (37 × 21 × 19 cm) provided by Tecniplast, in an air-conditioned room (temperature 21 ± 1 °C, relative humidity 60 ± 10%) under a reversed 12/12 h light/dark cycle with lights off from 07:00 a.m. to 07:00 p.m. Pellet food (Altromin-R 1320 maintenance diet for rats and mice, Rieper, Italy, see Supplementary Material for the product data sheet) and fresh tap-water were continuously available. In conformity with the European Directive, each cage was enriched with a paper towel (Celtex s.p.a., Lucca, Italy). All experiments were approved by the Italian Ministry of Health and carried out according to 2010/63/EU and the Italian legislation on animal experimentation, D.Lgs.vo 26/2014.

### 2.2. Treatment Administration

Rosmarinic acid (RA) was provided as powdered dry extract by AnalytiCon Discovery (Hermannswerder Haus, Potsdam, Germany). Both 3- and 24-month-old mice received 500 mg/Kg of RA dissolved in a pH 4 KH_2_PO_4_/K_2_HPO_4_ buffer in drinking bottles; this route of administration was selected due to its non-invasiveness. Within each group of age, control subjects received a pH 4 KH_2_PO_4_/K_2_HPO_4_ buffered water to control for the vehicle administration (see Supplementary Materials for further details). Bottles were weighed twice a week (when fresh solutions were prepared to avoid changes in pH and the proliferation of microorganisms since RA is unstable and cannot undergo autoclaving) to monitor the individual average daily consumption of solutions. The treatment was administered for 52 days. The dose and the overall length of treatment were selected based upon preliminary data from M. Ristow laboratory. The selected dose was equivalent to 38.8 mg/Kg for an average 60 Kg woman and to 37.4 mg/Kg for an average 80 Kg man (see [20] for calculation methods details). Subjects were allocated into the experimental groups based on a minimization approach avoiding body weight bias [21]. The final number of mice in the 24-month-old group was RA: males = 7, females = 7; Control: males = 5, females = 8 and reflects the natural survival curve of old subjects, with a reduced number of males compared to females (see also paragraph above).

### 2.3. Experimental Procedures

All tests were conducted during the dark phase (between 9 a.m. and 2 p.m.). Body weight of all subjects was assessed before starting the compound administration and after 28 (half of time length administration) and 50 days on treatment (before glucose metabolism assessments). Following 41 days on treatment, the emotional profile was assessed through the Elevated Plus Maze (EPM) test and the cognitive profile was assessed by means of the Morris Water Maze (MWM) test (days 43–45). Experimental subjects’ performance was recorded with a video camera (DCR-SX21E, Sony) and the behavioural analysis was performed by an observer blind to the experimental conditions using dedicated video tracking software (The Observer XT10 and Ethovision 1.9, Noldus software (Wageningen, The Netherlands).

**Figure 1:**
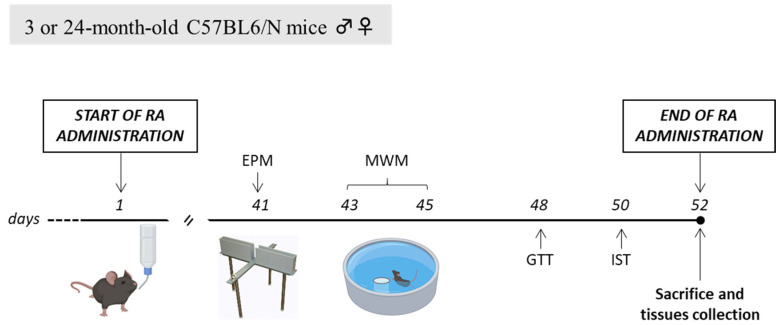
Experimental timeline. EPM, Elevated Plus Maze; MWM, Morris Water Maze; GTT, Glucose Tolerance Test; IST, Insulin Sensitivity Test.

Glucose homeostasis was evaluated by means of Glucose Tolerance Test (GTT, day 48) and Insulin Sensitivity Test (IST, day 50). We ensured a sufficient interval was present between subsequent tests to avoid carryover effects. At the end of behavioural and metabolic characterisation (day 52), mice were sacrificed by cervical dislocation followed by decapitation, and hippocampi from both hemispheres as well as trunk blood were collected and stored at −80 °C until the biochemical analyses. The experimental protocol is represented in Figure 1.

### 2.4. Emotional Profile and Cognitive Abilities

#### 2.4.1. Elevated Plus Maze (EPM)

Emotional profile and anxiety-like behaviours were evaluated through the EPM. The Plexiglas apparatus (dim grey floor, transparent walls) was made of two open (30 × 5 cm) and two closed arms (30 × 5 × 15 cm) extending from a central platform (5 × 5 cm) and raised to a height of 60 cm above the floor. Mice were individually placed on the central platform facing an open arm and allowed to freely explore the maze for a 5 min session. The time spent in the open versus the closed arms ((open/(open + closed)) × 100 and (closed/(open + closed)) × 100) as well as latency and frequency of arm entries and crossings (number of times the animal crosses the two imaginary lines on each arm) were used as a measures of anxiety levels. In addition, latency, frequency and duration of vertical exploration (rearing and wall-rearing: standing on the hind paws leaning or not on the wall), head-dipping (exploratory movement of head/shoulders over the side of maze) and stretched-attend posture (SAP, risk assessment behaviour in which the body is stretched forward and then retracted to the original position without any forward locomotion) were assessed [22].

#### 2.4.2. Morris Water Maze (MWM)

The MWM is a behavioural test used to evaluate spatial memory in rodents [23]. The apparatus used was a Plexiglas circular pool of 88 cm in diameter and 33 cm in height, filled with water (24–26 °C) stained with non-toxic white dye. The apparatus was ideally divided in four quadrants; a plastic platform (8 cm in diameter) was placed 10 cm from the edge of the pool in the target quadrant. Visual cues were stuck on the walls of the experimental room. Given the old age of experimental subjects, the learning protocol was specifically designed to assess possible age-related blindness and to reduce the anxiety due to the novel stressful environment and, at the same time, to avoid tiredness. Thus, the final protocol was based upon a modified version from the study from [24]. Briefly, mice were trained to learn the position of the platform in a 2-day training phase. On day 1 (habituation), the platform was always visible and tagged by a coloured flag. Animals were placed on the platform and were allowed to habituate to the experimental setting for 1 min; immediately after, they were gently put on water and underwent a first training trial (T1). After T1, two more trails (T2 and T3) took place and the start position changed on each trial. On day 2 (acquisition), the flag was removed and the platform was hidden 0.5 cm below the water surface; mice were given 3 training trials (T1, T2, T3) to locate the platform position. Trials were spaced by a 1 h inter-trial interval and the cut-off was set at 60 s for each trial. On experiment day 3, all subjects were tested for memory retention in the probe trial (60 s), during which the platform was removed from the pool and the time spent in the target quadrant was scored as a reliable measure of memory retention. During the acquisition phase, the latency to reach the platform was scored while time spent in each quadrant was assessed during the probe trial.

### 2.5. Glucose Homeostasis

Glycaemia was measured by means of a commercial glucometer (AccuCheck, Roche); blood was collected from the tail vein by means of the minimally invasive technique of tail-nick. For methodological details, see [25].

#### 2.5.1. Glucose Tolerance Test (GTT)

After an overnight food deprivation (15 h from 06:30 p.m. until 09:30 a.m.), blood glucose was assessed immediately before (0-baseline) and 30, 60, 120, 180 min after an intraperitoneal injection of 10% D-glucose solution (Sigma, St. Louis, MO, USA), at a dose of 2 g/kg.

#### 2.5.2. Insulin Sensitivity Test (IST)

After 5 h of food deprivation (from 09:30 a.m. until 02:30 p.m.), blood glucose was measured immediately before (0-baseline) and 15, 30, 60, and 120 min after an intraperitoneal injection of a 0.4 U/kg solution of human recombinant insulin (Humulin, Eli-Lilly, 100 U/mL).

### 2.6. Biochemical Analysis on Trunk Blood for the Evaluation of the Glucose and Fat Metabolism

At sacrifice, trunk blood was collected both in heparinised vials (for plasma separation) as well as in standard vials (not heparinised) to allow clotting for serum separation. Samples were collected and stored at −80 °C until they were analysed. Plasma levels of glucose, triglycerides (TAG), free fatty acids (FFA), fructosamine, total cholesterol (CHOL, both HDL and LDL), and high- and low-density lipoprotein (HDL and LDL, respectively) were analysed through automatic analyser (Cobas Mira S, Hoffmann-La Roche, Basel, Switzerland), using suitable commercial reagent kits (glucose HK, fructosamine, TAG, FS, cholesterol PAP, HDL-D, LDL-D; Diatools, Villmergen, Switzerland; and NEFA HR, Wako, Neuss, Germany). Serum concentrations of insulin, leptin and adiponectin were measured through immunoassay kit (MSD Mouse/Rat Insulin Kit, MSD Mouse Adiponectin Kit, MSD Mouse Leptin Kit) and were interpreted through a Sector Imager (Meso Scale Discovery, Gaithersburg, MD, USA) according to the instructions provided by the producer. These analyses were performed at the laboratory of Prof. Michael Ristow (Department of Health Sciences and Technology, ETH, Zurich).

### 2.7. Biochemical Analysis on the Hippocampus for the Evaluation of BDNF Levels

Hippocampal levels of BDNF were analysed through the enzyme-linked immunosorbent assay (ELISA) test using the “Mature BDNF Rapid ELISA” (Boisensis, West Thebarton Rd, Thebarton 5031, SA, Australia). Hippocampal samples (both ventral and dorsal, stored at −80 °C) were incubated with RIPA buffer (150 mM NaCl + TrisHCl 100 mM, pH = 8 + Triton X-1001%) and one tablet of protease inhibitors (Complete Mini EDTA Free Protease Inhibitor Cocktail Tablets, Roche) for 30 min at 4 °C. Successively, samples were homogenized through sonication and then centrifuged at 10,000 rpm for 10 min (4 °C). The supernatants were collected and used to BDNF measure. Samples, diluted mature BDNF standards, quality control sample and blank were placed on the pre-coated (mouse monoclonal anti-mature BDNF antibody) microplate wells and incubated on a shaker for 45 min. Successively, 5 washes were performed, and the plate was incubated with the detection antibody on a shaker for 30 min. After a further washing, the plate was incubated with the streptavidin-HRP conjugate for 30 min. Another washing was performed and, then, the substrate TMB was added into each well and the plate was incubated for 4–8 min without shaking in the dark. The TMB produced a colorimetric reaction (directly proportional to the concentration of BDNF; visible blue colour) that changed to yellow when the reaction was blocked by adding the stop solution. Finally, the reading of the absorbance at 450 nm was carried out through a spectrophotometer (Dynatech MR 5000, Dynatech Laboratories, Chantilly, VA, USA). To evaluate the concentration of BDNF, a standard curve with BDNF standard concentration on the *x*-axis and the optical density (OD) at 450 nm on the *y*-axis was plotted.

### 2.8. Statistics

Each mouse was defined as the experimental unit. All data were checked for normal distribution (Sphericity test) and successively analysed separately for 3- and 24-month-old mice using a one-way parametric analysis of variance (ANOVA) with treatment (vehicle vs. RA) as between-subjects’ factors and repeated measure as within-subjects’ factors (body weight, zones of the apparatuses (probe trial of the MWM: target vs. mean other quadrants; EPM: open vs. closed arms), time (GTT, IST, BW), trials/sessions (MWM)). The choice to carry-out separate analyses for 3- and 24-month-old subjects was aimed at preventing an obvious effect of age that would have masked the ability of RA to counteract the cognitive decline of old subjects. In fact, since the MWM protocol was specifically adapted to old mice, younger subjects were characterized by an outperformance in this test (see also Discussion). Since a main effect of sex was found on most of the parameters taken into account when performing a preliminary two-way ANOVA to better characterize the effects of RA treatment, male and female subjects were analysed separately. For ex vivo parameters, raw data were transformed to a percentage change compared to the appropriate control (either male or female). Grubb’s test (using 5% significance level critical value [26]) was used to detect outliers in the case of data following a normal distribution. The Tukey’s test was used to perform post hoc comparisons following ANOVA analysis when interaction effects were observed between treatment and repeated measures (graphically indicated with stars *). A level of *p* < 0.05 was chosen as statistically significant. Analysis was performed using GraphPad Prism 9 (Dotmatics, San Diego, CA, USA). Data were presented graphically as means ± SEM.

## 3. Results

### 3.1. Emotional Profile and Cognitive Abilities

#### 3.1.1. Elevated Plus Maze (EPM)

Old mice—All males showed a preference for the closed arms of the maze, and a similar trend was observed in females (main effect of zone, males: F(1,8) = 19.464; *p* = 0.0023; females: F(1,12) = 4.191; *p* = 0.0632). RA treatment did not affect this parameter (zone × treatment interaction: males: F(1,8) = 0.092; *p* = 0.7697; females: F(1,12) = 1.769; *p* = 0.2083). Moreover, RA treatment did not affect the latency, frequency and duration of the following parameters in either males or females: head-dipping (males: F(1,8) = 1.114, 3.918, 0.928; *p* = 0.3221, 0.0831, 0.3636; females: F(1,12) = 0.384, 0.007, 0.335; *p* = 0.5469, 0.9367, 0.5735), vertical exploration (males: F(1,8) = 0.470, 0.130, 0.513; *p* = 0.5125, 0.7277, 0.4941; females: F(1,12) = 3.138, 3.744, 1.889; *p* = 0.1018, 0.0769, 0.1945), SAP (males: F(1,8) = 0.191, 1.307, 1.417; *p* = 0.6734, 0.2861, 0.2681; females: F(1,12) = 0.107, 0.808; *p* = 0.7497, 0.3863), and crossing (males: F(1,8) = 0.148, 0.026; *p* = 0.7107, 0.8748; females: F(1,12) = 0.043, 2.549; *p* = 0.8393, 0.1363). Interestingly, although no effect of RA was observed on the latency to enter the closed arms on both males and females (males: F(1,8) = 0.398; *p* = 0.5455; females: F(1,12) = 0.623; *p* = 0.4454), a main effect of RA indicated a reduced number of visits to the closed arms in females only (males: F(1,8) = 1153; *p* = 0.3143; females: F(1,12) = 6.075; *p* = 0.0298). See Supplementary Figure S2.

Young mice—Both males and females showed a strong preference for the closed arms of the maze (main effect of zone, males: F(1,18) = 94.195; *p* < 0.0001; females: F(1,21) = 99.253; *p* < 0.0001). No effect of RA was observed on the percent time spent in the open vs. closed arms (zone × treatment interaction: males: F(1,18) = 1.816; *p* = 0.1945; females: F(1,21) = 0.499; *p* = 0.4875) or on the latency, frequency and duration of the following parameters in either males or females: head-dipping (males: F(1,18) = 0.907, 0.228, 0.505; *p* = 0.3536, 0.6385, 0.4865; females: F(1,21) = 0.242, 0.220, 0.001; *p* = 0.6280, 0.6439, 0.9718), vertical exploration (males: F(1,18) = 0.009, 0.005, 0.711; *p* = 0.9263, 0.9445, 0.4101; females: F(1,21) = 0.038, 0.841, 0.811; *p* = 0.8475, 0.3696, 0.3781), SAP (males: F(1,18) = 0.965, 0.003 0.003; *p* = 0.3389, 0.9563, 0.9595; females: F(1,21) = 0.017, 0.040, 0.299; *p* = 0.8983, 0.8427, 0.5900), and crossing (males: F(1,18) = 0.065, 0.000; *p* = 0.8014, 1; females: F(1,21) = 0.071, 0.008; *p* = 0.7930, 0.9311). No effect of RA was observed on the latency and frequency to enter the closed arms (males: F(1,18) = 0.088, 0.034; *p* = 0.7704, 0.8553; females: F(1,21) = 1.791, 0.023; *p* = 0.1951, 0.8808). See Supplementary Figure S2.

#### 3.1.2. Morris Water Maze (MWM)

**Figure 2:**
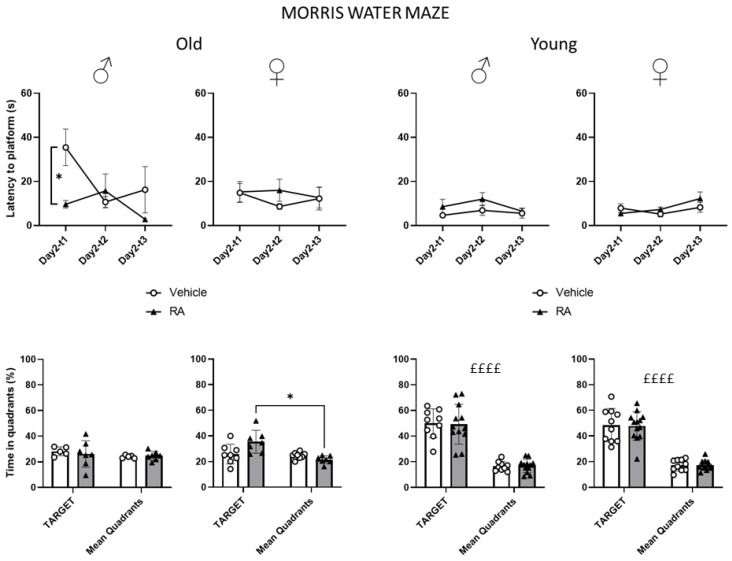
Morris Water Maze test. RA decreased the latency to reach the platform during T1 of acquisition in old males and increased the time spent in the target quadrant, compared to the other zones, during the probe trial in old females. All young mice learned and remembered the platform location regardless of RA treatment. Data are expressed as mean ± SEM. White dots and black triangles represents individual observations for Veh and RA mice, respectively. * *p* < 0.05 Tukey’s test RA-T1 vs. Veh-T1; RA-target vs. RA-others; ££££ *p* < 0.0001 main effect of zone Target vs. Mean quadrants; Veh-target vs. Veh-others; old: N = 5–8/group; young: N = 9–13/group.

Old mice—All subjects showed a reduction in the latency to reach the platform over time during the habituation phase (day 1, main effect of trials males: F(2,20) = 5.469; *p* = 0.0127; females: F(2,26) = 3.892; *p* = 0.0332). No difference due to RA treatment was observed during this phase (trials × treatment interaction, males: F(2,20) = 0.831; *p* = 0.4500; females: F(2,26) = 0.079; *p* = 0.9246). By contrast, during the acquisition phase (day 2), RA-treated male mice showed an increased ability to locate the hidden platform over trials (trials × treatment interaction F(2,20) = 3.513; *p* = 0.0493; post hoc comparisons RA-T1 vs. Veh-T1: *p* < 0.05) (Figure 2). RA did not affect the performance of female subjects during the acquisition phase (trials × treatment interaction: F(2,26) = 1.003; *p* = 0.3807). Analysis of the probe trial revealed that RA increased the ability to remember the platform location in females (zone × treatment interaction: F(1,13) = 5.012; *p* = 0.0433; post hoc comparison RA-target vs. RA-others: *p* < 0.05; Figure 2) while RA did not affect the memory retention of old males (zone × treatment interaction: F(1,10) = 0.175; *p* = 0.6842).

Young mice—All subjects showed a reduction in the latency to reach the platform over time during the habituation phase (main effect of trials: males: F(2,38) = 9.989; *p* = 0.0003; females: F(2,42) = 18.584; *p* < 0.0001). No difference due to RA treatment was observed during this phase (trials × treatment interaction: males: F(2,38) = 0.628; *p* = 0.5391; females: F(2,42) = 0.486; *p* = 0.6187). Regardless of RA treatment, all subjects learned to locate the hidden platform during the acquisition phase (trials × treatment interaction: males: F(2,38) = 0.310; *p* = 0.7355; females: F(2,42) = 1.717; *p* = 0.1920) and remembered the location of the platform in the probe trial (main effect of zone: males: F(1,19) = 64.945; *p* < 0.0001; females: F(1,21) = 84.027; *p* < 0.0001; Figure 2).

### 3.2. Hippocampal BDNF Levels

Old mice—When we analysed hippocampal BDNF levels, no effect of RA was found for both male and female mice (males: F(1,10) = 0.112; *p* = 0.7451; females: F(1,13) = 0.790; *p* = 0.3902). See Supplementary Figure S3.

Young mice—As for males, RA did not affect hippocampal BDNF levels (F(1,19) = 1.040; *p* = 0.3207). By contrast, a significant reduction in BDNF levels was observed in RA-treated females (F(1,21) = 17.096; *p* = 0.0005). See Supplementary Figure S3.

### 3.3. Metabolic Characterisation

#### 3.3.1. Body Weight

Old mice—A main effect of repeated measures (days) revealed that all subjects, regardless of sex or treatment, were characterised by a decrease in body weight over time, particularly at day 50 (males: F(2,20) = 17.360; *p* < 0.0001; females: F(2,26) = 10.740; *p* = 0.0004, post hoc comparisons day 50 vs. day 0 and day 28, *p* < 0.01 for both males and females). See Supplementary Figure S4.

Young mice—As for males, a main effect of repeated measures (days) showed that all subjects, regardless of treatment, increased their body weight over time (F(2,38) = 113.300; *p* < 0.0001). By contrast, a significant interaction between treatment and time showed that RA females were characterised by an increased in body weight on day 28 (F(2,42) = 7.301; *p* = 0.0019; post hoc comparison RA day 0 vs. RA day 28, *p* < 0.05 and RA day 28 vs. Veh-day 28: *p* < 0.01), while a significant increase in body weight was observed in the control females at the end of treatment (Veh day 28 vs. Veh day 50, *p* < 0.05). See Supplementary Figure S4.

#### 3.3.2. Glucose Tolerance Test (GTT)

Old mice—No effect of RA was found in males (interaction time × treatment: F(4,40) = 0.632; *p* = 0.6426) while a significant interaction between time and treatment (F(4,52) = 2.699; *p* = 0.0405) showed an overall slight trend to reduce glycaemia in RA-females (although post hoc comparison missed statistical significance). From the analysis of the basal values, we found that RA significantly reduced glycaemia at T0 only in males (males: F(1,10) = 6.016; *p* = 0341; females: F(1,13) = 1.759; *p* = 0.2076). See Supplementary Figure S5.

Young mice—RA-treated males were characterised by a strong trend towards significantly higher glycaemia (F(4,76) = 2.451; *p* = 0.0532). When post hoc comparisons were carried out [27], RA males showed higher values of glycaemia at T60 compared to controls (T60-RA vs. T60-Veh: *p* < 0.01); RA treatment did not affect females glycaemia (time × treatment interaction: F(4,84) = 1.216; *p* = 0.3102). By contrast, analysis of the basal values revealed that RA females had higher glycaemia while no difference was found in males (females: F(1,21) = 7.509; *p* = 0.0123; males: F(1,19) = 1.228; *p* = 0.2816). See Supplementary Figure S5.

#### 3.3.3. Insulin Sensitivity Test (IST)

**Figure 3:**
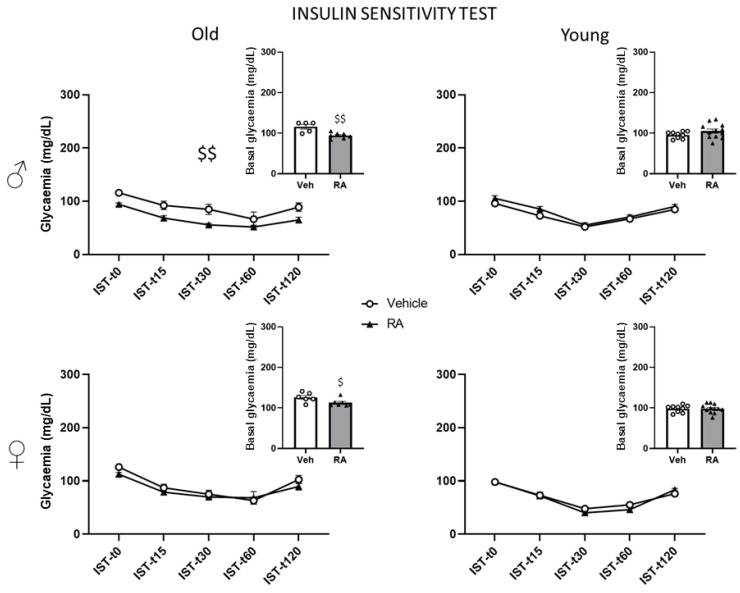
Insulin Sensitivity Test. RA decreased glycaemia values in old males, while no effect was observed in young subjects. Data are expressed as mean ± SEM. White dots and black triangles represents individual observations for Veh and RA mice, respectively. $ *p* < 0.05, $$ *p* < 0.01 main effect of treatment; old: N = 5–8/group; young: N = 9–13/group.

Old mice—Overall, RA resulted in reduced levels of glycaemia in male mice (main effect of treatment: F(1,10) = 15.497; *p* = 0.0028). No effect of RA was found in females from the analysis of the time course (time × treatment interaction: F(4,44) = 0.658; *p* = 0.6242; Figure 3). Interestingly, analysing the basal values, both male and female RA-treated mice resulted in reduced glycaemia (males: F(1,10) = 14.137; *p* = 0.0037; females: F(1,11) = 5.441; *p* = 0.0397).

Young mice—No effect of RA was found from the analysis of the time course in both males and females (time × treatment interaction: males: F(4,76) = 0.533; *p* = 0.7116; females: F(4,76) = 1.957; *p* = 0.1096), as well as from the analysis of the basal values (males: F(1,19) = 2.190; *p* = 0.1553; females: F(1,19) = 0.001; *p* = 0.9755). See Figure 3.

#### 3.3.4. Serum Parameters

Old mice—No effect of RA was found on insulin and adiponectin levels in males or females (males: F(1,9) = 0.247, 0.882; *p* = 0.6309, 0.3723; females: F(1,14) = 0.500, 0.856; *p* = 0.4910, 0.3705); leptin levels were also not affected by RA administration (males: F(1,10) = 0.091; *p* = 0.7696; females: F(1,13) = 0.989; *p* = 0.3381). See Supplementary Figure S6.

**Figure 4:**
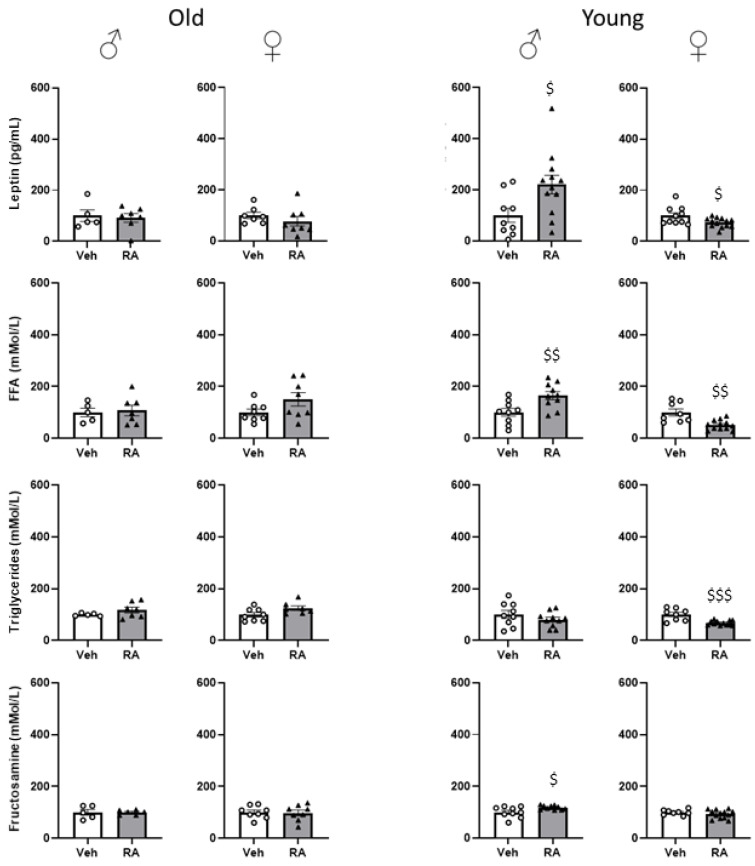
Serum and plasma parameters. RA treatment did not affect the lipid and glucose profile in old mice while it increased serum levels of leptin and plasma levels of FFAs and fructosamine in young males and decreased leptin, FFAs and TAG in young females. Data are expressed as mean ± SEM. White dots and black triangles represents individual observations for Veh and RA mice, respectively. $ *p* < 0.05, $$ *p* < 0.01, $$$ *p* < 0.001 main effect of treatment; old: N = 5–8/group; young: N = 9–13/group.

Young mice—No effect of RA was observed on the levels of insulin (males: F(1,19) = 0.064; *p* = 0.8028; females: F(1,18) = 1.201; *p* = 0.2875) and of adiponectin (males: F(1,16) = 1.643; *p* = 0.2181; females: F(1.21) = 0.476; *p* = 0.4979). Interestingly, RA affected circulating leptin levels in a sex-dependent fashion resulting in increased values in males (F(1,19) = 6.100; *p* = 0.0232) and decreased values in females (F(1,21) = 5.489; *p* = 0.0291; see Figure 4). See Supplementary Figure S6.

#### 3.3.5. Plasma Parameters

Old mice—RA treatment did not affect levels of FFA, total CHOL, HDL (males: F(1,10) = 0.069, 0.253, 0.448; *p* = 0.7980, 0.6258, 0.5185; females: F(1,14) = 3.050, 0.030, 0.0004; *p* = 0.1026, 0.8640, 0.9830), LDL (males: F(1,10) = 0.809; *p* = 0.3896; females: F(1,11) = 0.516; *p* = 0.4876), TAG, glucose (males: F(1,10) = 1.784, 0.182; *p* = 0.2113, 0.067; females: F(1,13) = 4.349, 0.124; *p* = 0.0573, 0.7306) and fructosamine (males: F(1,9) = 0.0004; *p* = 0.9829; females: F(1,14) = 0.030; *p* = 0.8660) in males or females. See Supplementary Figure S6.

Young mice—RA did not affect levels of total CHOL in males or females (males: F(1,16) = 0.925; *p* = 0.3506; females: F(1,19) = 3.252; *p* = 0.0873). By contrast, only RA females were characterised by decreased levels of HDL (females: F(1,19) = 5.807; *p* = 0.0263; males: F(1,16) = 0.002; *p* = 0.9681) and increased levels of LDL (females: F(1,19)= 8.508; 0.0088; males: F(1,17)= 0.357; *p* = 0.5580). Moreover, sex-dependent effects were found on other parameters, with RA males showing increased levels of FFA and of fructosamine (F(1,17) = 8.882, 6.231; *p* = 0.0084; 0.0231, respectively for FFAs and fructosamine) while females were characterised by decreased levels of both FFA and TAG (females: F(1,18) = 14.537, 18.957; *p* = 0.0013, 0.0004, respectively for FFA and TAG). No effect was observed with regard to TAG in males (F(1,17) = 1.317; *p* = 0.2670) and fructosamine in females (F(1,19) = 1.088; *p* = 0.3101). In addition, RA females showed a significant reduction in plasma glucose levels, an effect that was not observed in males (females: F(1,19) = 8.258; *p* = 0.0097; males: F(1,17) = 0.520; *p* = 0.4806). See Figure 4 and Supplementary Figure S6.

## 4. Discussion

Our study supports the notion that oral administration of RA increases some features of healthspan in a mouse model of physiological aging. By contrast, its administration in young adult mice disrupts the metabolic profile in a sex-dependent fashion, suggesting caution in its administration at younger ages.

RA enhanced the learning and memory in 24-month-old mice, an effect that was associated with an improved ability to maintain glucose homeostasis (see below). In particular, while RA-males showed a decrease in the latency to reach the platform during the first trial of the acquisition phase, RA-females spent more time in the target quadrant during the probe trial, overall suggesting that RA might improve learning abilities in males and potentiate memory retention in females. This latter effect was also associated to a decrease in the frequency to enter the closed arms of the EPM only in RA-females, possibly suggesting a reduced anxiety-like behaviour. When we investigated whether the effects in the cognitive and emotional profile might be due to changes in the levels of the BDNF—a neurotrophin playing a pivotal role in brain plasticity [17]—no change was observed in its hippocampal protein levels as a result of RA treatment. Likewise, no effect of RA administration was observed in peripheral blood metabolic markers. However, basal glycaemia levels were decreased upon fasting in male and female subjects. Moreover, in the GTT, RA-females showed a lower increase in levels of glycaemia following the glucose injection while male mice appeared to be more sensitive to an exogenous insulin challenge following RA treatment.

The impact of glucose homeostasis on brain functions appears to be so meaningful that pathologies such as Alzheimer’s Disease (AD) have been defined as Type 3 Diabetes [28,29,30,31]. Peripheral insulin resistance characterising Type 2 Diabetes and excessive adiposity may greatly contribute to the neuroinflammation and insulin resistance observed in the brain by increasing the production of cytotoxic lipids crossing the blood–brain barrier [16]. Given this evidence, it is possible to hypothesize that the observed effect of RA on the cognitive performance in old subjects may be due, at least in part, to peripheral mechanisms related to a more effective management of glucose metabolism possibly through its anti-inflammatory and antioxidant properties [1,32]. This peripheral mechanism is worthy of attention when considering that a reduction in physical health may modulate and synergise with mental outcomes reinforcing one another in a vicious cycle, having a strong impact on the quality of life during aging by reducing the overall healthspan [1,4].

When the behavioural profile of young mice was scored, as expected, no difference was observed as a result of RA treatment since both males and females showed a clear preference for the closed arms of the EPM and properly learned and remembered the platform location in the MWM. In this context, it must be emphasised that the MWM protocol was specifically adapted to assess the cognitive abilities of old mice who are generally characterised by poor sensorimotor performance and increased anxiety-related behaviours when compared to young adults. Moreover, we cannot exclude that the overall composition of the diet might also have played a role in the observed effects ([33] see also Supplementary Materials). In this case, we can hypothesize that the standard antioxidant compounds that are regularly added to the diet might have blunted the effects of RA.

Our data suggest that RA treatment worsened glucose homeostasis in young subjects, particularly in males, who also showed increased levels of blood fructosamine, a marker of impaired glucose homeostasis [34]. Young males were also characterised by increased FFA and leptin levels. Elevated blood glucose levels and FFA represent a form of metabolic overload often observed in pre-diabetic conditions also found in association with altered leptin levels, an adipose tissue-derived hormone involved in the modulation of both insulin sensitivity and secretion [35,36]. Thus, we can hypothesize that RA treatment, at least in young males, might disrupt glucose homeostasis.

Energy metabolism is finely tuned in mammals to meet sex-specific and evolutionary conserved needs possibly related to gestation and lactation in females, whereas the male metabolism may reflect a steady state. This consideration might explain why we observed opposite sex-dependent modulatory effects of RA leptin and FFA. In general, females show increased lipids storage and better insulin sensitivity while males are characterised by greater visceral adiposity and lipid oxidation, all effects orchestrated by sex chromosomes and hormones [37,38]. In addition to buffering reactive oxygen species (ROS) a growing number of evidence suggests powerful effects of RA in the modulation of aromatase, the enzyme responsible for the biosynthesis of estradiol and estrone from androgens [39]. Our data are in line with the study from Zich and co-workers that reported beneficial effects of RA in reducing CHOL and TAG in oestrogen-deficient female rats, a model for menopause, supporting a role for RA, or its metabolites, to increase aromatase activity (possibly by preferentially targeting subcutaneous fat) and to act as a powerful antioxidant [40]. However, in association to the decrease in FFA, leptin and TAG we also found a specific decrease in HDL cholesterol and, most importantly, in hippocampal BDNF levels (this latter effect was not observed in young male mice). Sex hormones may modulate leptin levels [41,42,43] and recent evidence suggest that leptin may regulate the transcription of the *Bdnf* gene in the hippocampus through a positive feedback regulation [44]. Thus, it is possible to hypothesize that RA by disrupting lipid metabolism in females might in turn negatively affect central mediators of brain plasticity [45].

It is also possible to hypothesize that the discrepancies observed in the effects of RA administration in old and young subjects might rely not only upon a different hormonal milieu, but also upon age-related redox balance. In fact, while OS may precipitate health outcomes in the aging process and plays a pivotal role in inflammaging [46], under physiological conditions ROS are necessary in many vital processes such as insulin signalling. To this regard, we have previously shown that decreasing ROS levels, by means of either exogenous antioxidants or by genetically modifying the redox environment, resulted in beneficial effects on glucose and fat homeostasis in murine models of high-fat diet nutrition during foetal life while leading to conflicting and sex-specific effects in control subjects [25,47]. Ultimately, these data indicate that decreasing the ROS level when unnecessary, as in young healthy subjects, might negatively affect metabolic signalling pathways, resulting in sex-specific outcomes.

## Data Availability

The data presented in this study are available in Supplementary Materials. Raw data are available on request from the corresponding authors.

## Supplementary Materials

The following supporting information can be downloaded at: https://www.mdpi.com/article/10.3390/nu15153366/s1. Supplementary Materials: S1. Pilot study to determine RA solubility in water and solution palatability; S2. Evaluation of RA toxicity; S2.1 Biochemical analysis to determine plasmatic ALAT concentration; S2.2 Results; S3. Sample size determination; S4. Minimization of potential confounders; S5. Exclusion criteria and summary statistics; Supplementary figures. ARRIVE checklist. Diet data sheet.
