# Supplementary material for: Rosmarinic Acid Improves Cognitive Abilities and Glucose Metabolism in Aged C57Bl/6N Mice While Disrupting Lipid Profile in Young Adults in a Sex-Dependent Fashion
Source: Nutrients. 2023 Jul 28;15(15):3366. doi: 10.3390/nu15153366 (PMC10421458; doi:10.3390/nu15153366)
Supplement: Supplementary file 1 [file nutrients-15-03366-s001.zip › supplemetary materials.pdf]

Supplementary Materials

1. Pilot study to determine RA solubility in water and solution palatability

Rosmarinic acid (RA) was extracted from edible plants (*Rosmarinus officinalis*) and provided as powdered dry extract by AnalytiCon Discovery (Hermannswerder Haus, Potsdam, Germany).

The dose of RA was 500 mg/Kg, selected by a previous study conducted from M. Ristow laboratory (Ristow et al., unpublished). In order to be able to administer RA through drinking water, a non-invasive route of administration, a preliminary study was necessary to evaluate average daily water intake for each mouse so to determine RA concentration. To this aim, water intake was monitored by weighing water bottles every Monday for 4 weeks. To obtain individual daily consumption, difference in weight was averaged among days and number of subjects/cage. Results showed an average daily consumption of 5.27 ml and 5.15 ml respectively for male and female mice. Thus, considering an average body weight of 30 g/mouse and an average daily consumption of about 5 ml, the calculation of concentration was as follows:

500 mg/Kg→500 mg/1000 g mouse→0.5mg/g mouse→15mg/mouse/day

15 mg/5 ml→3 mg/ml→750 mg in 250 ml (small mouse bottle)

RA powder showed good solubility in water and the solution obtained showed a good stability (no deposits or alterations of odor and color were detected).

Since the RA solution had a pH of 2.86 possibly affecting its palatability, a pilot study was carried out in order to select the pH buffer in which dissolve the RA powdered dry extract and the palatability of the buffered RA solution by mice. To this aim, a phosphate buffer solution (H_2KPO_4/K_2HPO_4) was selected since it was well tolerated, palatable and routinely used in the food industry. More in detail, 13.6 g of KH_2PO_4 were dissolved in 1 l of drinking water to obtain a 100 mM buffer (pH 5.8). Successively a 100 mM H_3PO_4 solution was added to obtain three different buffers at the following pH: 3, 4 or 5. The buffer solutions and the control (tap water) were administered to mice in the drinking bottles and the spontaneous consumption was monitored over 5 days by weighting the bottles every 24 hours. The consumption was comparable for the three solutions however, a slight preference was observed for the pH 4 buffer (pH 3, 5.19 ml/mouse, n=9; pH 4, 5.28 ml/mouse, n=8; pH 5, 5.17 ml/mouse, n=8; control, 4.58 ml/mouse, n=4) that was eventually selected as vehicle.

In order to test the palatability of the RA solution dissolved in the selected buffer, 1.5 g of RA was dissolved in 400 ml of KH_2PO_4 solution. Then, a K_2HPO_4 solution was added to adjust to pH 4 and KH_2PO_4 solution was added making up the volume of 500 ml. The solution was administered to 14 male mice (2/3 for cage) and the average daily individual consumption was monitored over 3 days and compared to the consumption of vehicle (phosphate buffer pH 4). Results indicated no significant differences between vehicle and RA solution consumption.

2. Evaluation of RA toxicity

To determine if RA 500 mg/Kg dose induced liver toxicity at old age, we measured plasma Alanine Aminotransferase (ALAT) levels, since an increase of this enzyme is considered as a key marker of hepatocellular injury. Blood samples from the tail vein (day 0) and from the trunk blood (at sacrifice, day 52) from a total of 16 mice (RA: 3 males, 5 females; Control: 3 males, 5 females) was collected in heparinized vials before and after 52 days of RA treatment (500 mg/Kg) and stored at -80°C until the biochemical analyses.

2.1. Biochemical analysis to determine plasmatic ALAT concentration

Determination of alanine-aminotransferase (ALAT) in plasma was performed using an automated analyser (Cobas Mira S, Hoffmann-La Roche, Basel, Switzerland) with commercially available reagent kit (ALAT (GPT) FS (IFCC mod.), diatools, Villmergen, Switzerland).

2.2. Results

No significant difference was observed due to RA treatment (repeated measures x treatment interaction, males: $F(1,4)=0.001$; $p=0.9811$; females: $F(1,8)=0.044$; $p=0.8393$, Fig. 1S), thus we can state that there was no toxicity.

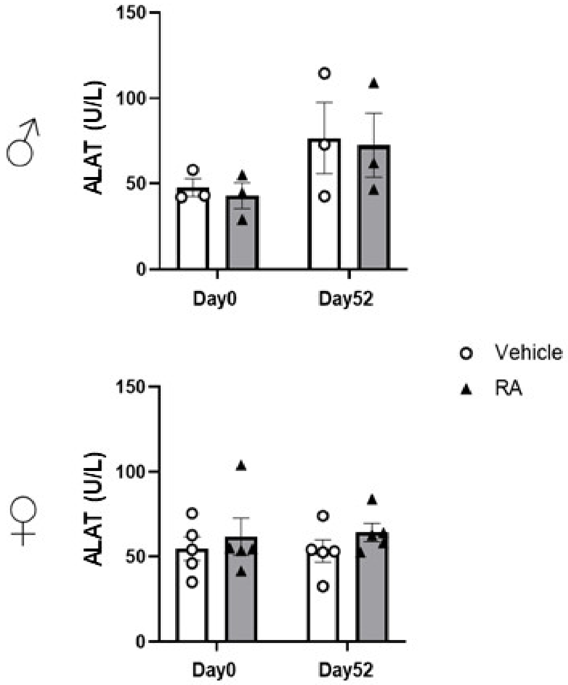

Figure S1. Alanine transaminase concentration. No significant difference was observed between RA and control mice at old age. Data are expressed as mean ± SEM. White dots and black triangles represents individual observations for Veh and RA mice, respectively. N=3-5/group.

3. Sample size determination

The sample size needed for each experiment was established as n=10 by performing a statistical power analysis to allow the assessment of a standardized difference between any pair of means of at least 0.65 SD for any response variable (with a medium to large effect sizes) based on the distribution of the primary response variable, the number of experimental groups for each pair of comparisons (age; sex), the experimental design (dependent or independent statistical units), with a level of significance alpha=0.05 in a two-tailed test and a power (1 – beta)=0.80. For this experiment, since we aimed at characterize the ability of RA to counteract cognitive decline of old subjects, the Morris Water Maze test was selected as primary response variable based on the available literature on senescent mice.

4. Minimization of potential confounders

Subjects were allocated into the experimental groups based on a minimization approach avoiding body weight bias. After the assignment to each experimental group, cages were allocated into the animal facility avoiding bias due to the cage position within the room. All tests were conducted during the dark phase (09:00 am – 02:00 pm) that is the period of maximum activity of nocturnal rodents. The order of animal testing as well as the starting position of each mouse for the Elevated Plus Maze test were counterbalanced considering sex and treatment.

5. Exclusion criteria and summary statistics

Elevated Plus Maze

Exclusion criteria: animals falling from the maze after 60 sec following the beginning of testing were excluded from the statistical analysis. The final number of mice in the 24-month-old group was RA: males=5, females=7; Control: males=5, females=7; the final number of mice in the 3-month-old group was RA: males=12, females=13; Control: males=8, females=10.

TIME IN ARMS (%)

Old males	Mean	SD	SE		Young males	Mean	SD	SE
Veh, open %	36.131	11.598	5.187		Veh, open %	26.084	8.804	3.113
Veh, close %	63.869	11.598	5.187		Veh, close %	73.916	8.804	3.113
RA, open %	37.912	6.210	2.777		RA, open %	31.915	9.888	2.854
RA, close %	62.088	6.210	2.777		RA, close %	68.085	9.888	2.854
**Old females**	**Mean**	**SD**	**SE**		**Young females**	**Mean**	**SD**	**SE**
Veh, open %	37.051	11.017	4.164		Veh, open %	27.504	12.984	4.106
Veh, close %	62.949	11.017	4.164		Veh, close %	72.496	12.984	4.106
RA, open %	47.250	17.036	6.439		RA, open %	24.069	10.357	2.872
RA, close %	52.750	17.036	6.439		RA, close %	75.931	10.357	2.872

CLOSED ARMS ENTRIES (N. OF EVENTS)

Old males	Mean	SD	SE		Young males	Mean	SD	SE
Veh	8.200	2.683	1.200		Veh	13.250	2.816	0.996
RA	9.600	1.140	0.510		RA	12.917	4.522	1.305
**Old females**	**Mean**	**SD**	**SE**		**Young females**	**Mean**	**SD**	**SE**
Veh	10.286	1.113	0.421		Veh	16.900	2.685	0.849
RA	7.143	3.185	1.204		RA	16.769	3.370	0.935

Morris Water Maze

Exclusion criteria: visual impairment as assessed in the cued phase (habituation) or overt physical impairment while swimming. The final number of mice in the 24-month-old group was RA: males=7, females=7; Control: males=5, females=8; the final number of mice in the 3-month-old group was RA: males=12, females=13; Control: males=9, females=10.

HABITUATION - Latency (s)								
**Old males**	**Mean**	**SD**	**SE**		**Young males**	**Mean**	**SD**	**SE**
Veh, day1-t1	25.376	20.107	8.992		Veh, day1-t1	30.342	18.964	6.321
Veh, day1-t2	18.064	14.803	6.620		Veh, day1-t2	10.369	7.581	2.527
Veh, day1-t3	11.704	8.800	3.936		Veh, day1-t3	12.716	18.117	6.039
RA, day1-t1	38.314	22.276	8.420		RA, day1-t1	28.767	13.452	3.883
RA, day1-t2	21.229	21.877	8.269		RA, day1-t2	13.773	18.406	5.313
RA, day1-t3	7.200	4.087	1.545		RA, day1-t3	4.963	2.407	0.695
**Old females**	**Mean**	**SD**	**SE**		**Young females**	**Mean**	**SD**	**SE**
Veh, day1-t1	34.840	22.677	8.018		Veh, day1-t1	27.724	11.284	3.568
Veh, day1-t2	18.920	18.423	6.513		Veh, day1-t2	10.396	10.629	3.361
Veh, day1-t3	14.255	7.443	2.631		Veh, day1-t3	7.556	4.471	1.414
RA, day1-t1	31.423	20.054	7.580		RA, day1-t1	27.880	16.088	4.462
RA, day1-t2	19.966	19.597	7.407		RA, day1-t2	15.348	16.278	4.515
RA, day1-t3	15.674	11.504	4.348		RA, day1-t3	5.643	4.792	1.329

ACQUISITION - Latency (s)								
**Old males**	**Mean**	**SD**	**SE**		**Young males**	**Mean**	**SD**	**SE**
Veh, day1-t1	35.440	18.589	8.313		Veh, day1-t1	4.604	2.217	0.739
Veh, day1-t2	10.680	5.549	2.482		Veh, day1-t2	6.858	6.907	2.302
Veh, day1-t3	16.248	23.489	10.505		Veh, day1-t3	5.547	6.596	2.199
RA, day1-t1	9.623	4.622	1.747		RA, day1-t1	8.500	11.774	3.399
RA, day1-t2	15.697	20.363	7.697		RA, day1-t2	11.953	10.076	2.909
RA, day1-t3	2.777	1.956	0.739		RA, day1-t3	6.483	4.737	1.367
**Old females**	**Mean**	**SD**	**SE**		**Young females**	**Mean**	**SD**	**SE**
Veh, day1-t1	14.845	11.862	4.194		Veh, day1-t1	7.852	6.225	1.978
Veh, day1-t2	8.645	3.616	1.279		Veh, day1-t2	5.172	3.959	1.252
Veh, day1-t3	12.245	14.779	5.225		Veh, day1-t3	8.224	7.161	2.264
RA, day1-t1	15.091	12.620	4.770		RA, day1-t1	5.480	3.872	1.074
RA, day1-t2	21.846	21.377	8.080		RA, day1-t2	7.274	4.003	1.110
RA, day1-t3	16.766	12.796	4.837		RA, day1-t3	12.218	10.784	2.991

PROBE - Time in quadrants (%)								
**Old males**	**Mean**	**SD**	**SE**		**Young males**	**Mean**	**SD**	**SE**
Veh, target %	28.120	3.431	1.534		Veh, target %	49.991	11.290	3.763
Veh, others %	23.917	1.154	0.516		Veh, others %	16.590	3.711	1.237
RA, target %	26.098	10.285	3.887		RA, target %	49.217	15.497	4.474
RA, others %	24.600	3.456	1.306		RA, others %	16.845	5.194	1.499
**Old females**	**Mean**	**SD**	**SE**		**Young females**	**Mean**	**SD**	**SE**
Veh, target %	25.858	7.627	2.697		Veh, target %	48.300	13.062	4.131
Veh, others %	24.710	2.552	0.902		Veh, others %	17.206	4.382	1.386
RA, target %	35.414	8.945	3.381		RA, target %	47.787	11.048	3.064
RA, others %	21.506	2.967	1.122		RA, others %	17.389	3.680	1.021

Hippocampal BDNF levels

Exclusion criteria: whether following the extraction procedure the amount of tissue available was not sufficient to assay BDNF levels, subjects were excluded from the statistical analysis. The final number of mice in the 24-month-old group was RA: males=7, females=7; Control: males=5, females=8; the final number of mice in the 3-month-old group was RA: males=12, females=13; Control: males=9, females=10.

BDNF (pg/mg)								
**Old males**	**Mean**	**SD**	**SE**		**Young males**	**Mean**	**SD**	**SE**
Veh	9.445	7.455	3.334		Veh	6.011	1.709	0.570
RA	10.793	6.478	2.448		RA	7.217	3.210	0.927
**Old females**	**Mean**	**SD**	**SE**		**Young females**	**Mean**	**SD**	**SE**
Veh	10.550	8.210	2.903		Veh	9.560	1.666	0.527
RA	15.338	12.488	4.720		RA	6.572	1.756	0.487

Body weight

Exclusion criteria for the analysis of this parameter were not set. The final number of mice in the 24-month-old group was RA: males=7, females=7; Control: males=5, females=8; the final number of mice in the 3-month-old group was RA: males=12, females=13; Control: males=9, females=10.

BODY WEIGHT (g)								
**Old males**	**Mean**	**SD**	**SE**		**Young males**	**Mean**	**SD**	**SE**
Veh, day 0	31.200	0.255	0.114		Veh, day 0	23.700	2.458	0.819
Veh, day 28	32.200	1.007	0.451		Veh, day 28	27.078	3.073	1.024
Veh, day 50	30.700	0.212	0.095		Veh, day 50	28.356	3.259	1.086
RA, day 0	32.243	2.501	0.945		RA, day 0	23.350	1.523	0.440
RA, day 28	32.614	2.756	1.042		RA, day 28	25.617	2.071	0.598
RA, day 50	30.100	1.945	0.735		RA, day 50	27.325	3.024	0.873
**Old females**	**Mean**	**SD**	**SE**		**Young females**	**Mean**	**SD**	**SE**
Veh, day 0	26.350	1.307	0.462		Veh, day 0	20.820	0.939	0.297
Veh, day 28	26.213	1.348	0.477		Veh, day 28	20.210	2.124	0.672
Veh, day 50	25.700	1.645	0.582		Veh, day 50	23.330	0.702	0.222
RA, day 0	26.014	1.979	0.748		RA, day 0	20.877	0.828	0.230
RA, day 28	26.643	1.764	0.667		RA, day 28	22.046	0.859	0.238
RA, day 50	25.100	1.443	0.546		RA, day 50	23.000	0.920	0.255

Glucose Tolerance Test

Exclusion criteria for the analysis of this parameter were not set. The final number of mice in the 24-month-old group was RA: males=7, females=7; Control: males=5, females=8; the final number of mice in the 3-month-old group was RA: males=11, females=13; Control: males=10, females=10.

GLUCOSE TOLERANCE TEST - Glycaemia (mg/dL)								
**Old males**	**Mean**	**SD**	**SE**		**Young males**	**Mean**	**SD**	**SE**
Veh, GTT-t0	131.800	11.432	5.113		Veh, GTT-t0	104.700	16.125	5.099
Veh, GTT-t30	187.200	59.655	26.678		Veh, GTT-t30	232.700	60.579	19.157
Veh, GTT-t60	152.800	14.789	6.614		Veh, GTT-t60	138.600	23.529	7.440
Veh, GTT-t120	128.200	15.881	7.102		Veh, GTT-t120	103.500	21.844	6.908
Veh, GTT-t180	109.600	15.931	7.125		Veh, GTT-t180	92.500	14.073	4.450
RA, GTT-t0	112.000	15.155	5.728		RA, GTT-t0	113.545	20.002	6.031
RA, GTT-t30	178.571	41.653	15.743		RA, GTT-t30	235.636	42.359	12.772
RA, GTT-t60	160.000	39.179	14.808		RA, GTT-t60	184.727	34.955	10.539
RA, GTT-t120	129.000	23.875	9.024		RA, GTT-t120	117.545	13.538	4.082
RA, GTT-t180	107.429	15.576	5.887		RA, GTT-t180	112.545	13.634	4.111
**Old females**	**Mean**	**SD**	**SE**		**Young females**	**Mean**	**SD**	**SE**
Veh, GTT-t0	105.500	31.473	11.128		Veh, GTT-t0	92.600	11.890	3.760
Veh, GTT-t30	188.500	45.482	16.080		Veh, GTT-t30	233.800	28.397	8.980
Veh, GTT-t60	151.500	36.465	12.892		Veh, GTT-t60	162.100	7.400	2.340
Veh, GTT-t120	128.625	20.570	7.273		Veh, GTT-t120	105.200	10.239	3.238
Veh, GTT-t180	114.625	17.386	6.147		Veh, GTT-t180	107.000	9.626	3.044
RA, GTT-t0	121.714	7.296	2.758		RA, GTT-t0	107.538	13.709	3.802
RA, GTT-t30	163.429	26.602	10.054		RA, GTT-t30	229.769	47.687	13.226
RA, GTT-t60	128.857	18.407	6.957		RA, GTT-t60	171.154	24.231	6.720
RA, GTT-t120	115.143	13.801	5.216		RA, GTT-t120	115.385	12.718	3.527
RA, GTT-t180	102.286	15.283	5.776		RA, GTT-t180	100.615	9.224	2.558

Insulin sensitivity test

Exclusion criteria: animals showing blood glucose levels ≤40 mg/dl after insulin challenge were excluded from the test and received an i.p. glucose injection to restore glucose homeostasis. The final number of mice in the 24-month-old group was RA: males=7, females=7; Control: males=5, females=6; the final number of mice in the 3-month-old group was RA: males=12, females=12; Control: males=9, females=9.

INSULIN SENSITIVITY TEST - Glycaemia (mg/dL)								
**Old males**	**Mean**	**SD**	**SE**		**Young males**	**Mean**	**SD**	**SE**
Veh, IST-t0	115.800	12.775	5.713		Veh, IST-t0	95.778	8.333	2.778
Veh, IST-t15	92.200	17.740	7.933		Veh, IST-t15	72.556	9.901	3.300
Veh, IST-t30	84.800	21.476	9.604		Veh, IST-t30	51.778	11.745	3.915
Veh, IST-t60	66.400	29.720	13.291		Veh, IST-t60	66.778	9.692	3.231
Veh, IST-t120	88.800	17.866	7.990		Veh, IST-t120	84.667	14.000	4.667
RA, IST-t0	94.143	7.244	2.738		RA, IST-t0	105.250	17.705	5.111
RA, IST-t15	68.286	12.379	4.679		RA, IST-t15	85.250	16.581	4.787
RA, IST-t30	55.714	7.544	2.851		RA, IST-t30	55.167	14.819	4.278
RA, IST-t60	51.571	9.554	3.611		RA, IST-t60	70.417	14.607	4.217
RA, IST-t120	64.857	13.409	5.068		RA, IST-t120	90.167	13.204	3.812
**Old females**	**Mean**	**SD**	**SE**		**Young females**	**Mean**	**SD**	**SE**
Veh, IST-t0	126.000	11.679	4.768		Veh, IST-t0	98.111	8.781	2.927
Veh, IST-t15	87.000	16.971	6.928		Veh, IST-t15	73.222	17.761	5.920
Veh, IST-t30	75.000	18.067	7.376		Veh, IST-t30	47.889	9.892	3.297
Veh, IST-t60	63.167	16.582	6.770		Veh, IST-t60	55.111	13.824	4.608
Veh, IST-t120	102.667	18.811	7.680		Veh, IST-t120	76.111	13.615	4.538
RA, IST-t0	112.571	9.090	3.436		RA, IST-t0	98.250	11.005	3.177
RA, IST-t15	78.857	12.941	4.891		RA, IST-t15	71.750	8.346	2.409
RA, IST-t30	69.857	15.910	6.014		RA, IST-t30	40.083	9.520	2.748
RA, IST-t60	68.429	31.149	11.773		RA, IST-t60	46.167	14.012	4.045
RA, IST-t120	89.714	19.024	7.190		RA, IST-t120	83.583	8.512	2.457

Serum parameters

Exclusion criteria: whether following the extraction procedure the amount of serum available was not sufficient to assay insulin, leptin and adiponectin levels, subjects were excluded from the statistical analysis. The final number of mice in the 24-month-old group was RA: males=6, females=7; Control: males=5, females=8; the final number of mice in the 3-month-old group was RA: males=12, females=10; Control: males=9, females=10 for insulin.

INSULIN (pg/mL)								
**Old males**	**Mean**	**SD**	**SE**		**Young males**	**Mean**	**SD**	**SE**
Veh	100.000	44.020	19.686		Veh	100.000	73.825	24.608
RA	114.694	52.317	21.358		RA	93.770	37.579	10.848
**Old females**	**Mean**	**SD**	**SE**		**Young females**	**Mean**	**SD**	**SE**
Veh	100.000	42.011	14.853		Veh	100.000	77.512	24.511
RA	124.687	89.324	31.581		RA	154.830	137.899	43.608

The final number of mice in the 24-month-old group was RA: males=7, females=7; Control: males=5, females=8; the final number of mice in the 3-month-old group was RA: males=12, females=13; Control: males=9, females=10 for leptin.

LEPTIN (pg/mL)								
**Old males**	**Mean**	**SD**	**SE**		**Young males**	**Mean**	**SD**	**SE**
Veh	100.000	50.890	22.759		Veh	100.000	82.070	27.357
RA	91.646	44.918	16.978		RA	220.017	126.801	36.604
**Old females**	**Mean**	**SD**	**SE**		**Young females**	**Mean**	**SD**	**SE**
Veh	100.000	32.832	12.409		Veh	100.000	35.070	11.090
RA	76.791	53.405	18.882		RA	73.440	18.672	5.179

The final number of mice in the 24-month-old group was RA: males=6, females=8; Control: males=5, females=7; the final number of mice in the 3-month-old group was RA: males=11, females=13; Control: males=7, females=10 for adiponectin.

ADIPONECTIN (pg/mL)								
**Old males**	**Mean**	**SD**	**SE**		**Young males**	**Mean**	**SD**	**SE**
Veh	100.000	22.163	9.912		Veh	100.000	36.673	13.861
RA	83.596	33.249	13.574		RA	125.211	42.897	12.934
**Old females**	**Mean**	**SD**	**SE**		**Young females**	**Mean**	**SD**	**SE**
Veh	100.000	35.926	12.702		Veh	100.000	28.377	8.974
RA	84.270	31.958	11.299		RA	106.159	13.583	3.767

Plasma parameters

Exclusion criteria: whether following the extraction procedure the amount of plasma available was not sufficient to assay glucose, fructosamine, total CHOL, HDL, LDL, FFA and TAG levels, subjects were excluded from the statistical analysis. The final number of mice in the 24-month-old group was RA: males=7, females=7; Control: males=5, females=8; the final number of mice in the 3-month-old group was RA: males=10, females=13; Control: males=9, females=8 for glucose.

GLUCOSE (mMol/L)								
**Old males**	**Mean**	**SD**	**SE**		**Young males**	**Mean**	**SD**	**SE**
Veh	100.000	14.161	6.333		Veh	100.000	27.675	9.225
RA	103.836	16.111	6.089		RA	107.297	15.902	5.029
**Old females**	**Mean**	**SD**	**SE**		**Young females**	**Mean**	**SD**	**SE**
Veh	100.000	20.264	7.164		Veh	100.000	18.869	6.671
RA	96.543	16.744	6.328		RA	79.124	14.358	3.982

The final number of mice in the 24-month-old group was RA: males=6, females=7; Control: males=5, females=8; the final number of mice in the 3-month-old group was RA: males=10, females=13; Control: males=9, females=8 for fructosamine.

FRUCTOSAMINE (mMol/L)								
**Old males**	**Mean**	**SD**	**SE**		**Young males**	**Mean**	**SD**	**SE**
Veh	100.000	25.016	11.188		Veh	100.000	21.291	7.097
RA	99.760	9.216	3.763		RA	118.070	8.120	2.568
**Old females**	**Mean**	**SD**	**SE**		**Young females**	**Mean**	**SD**	**SE**
Veh	100.000	24.384	8.621		Veh	100.000	10.105	3.573
RA	97.605	30.960	10.946		RA	93.188	16.581	4.599

The final number of mice in the 24-month-old group was RA: males=7, females=7; Control: males=5, females=8; the final number of mice in the 3-month-old group was RA: males=10, females=13; Control: males=8, females=8 for total CHOL.

TOTAL CHOLESTEROL (mMol/L)								
**Old males**	**Mean**	**SD**	**SE**		**Young males**	**Mean**	**SD**	**SE**
Veh	100.000	14.106	6.309		Veh	100.000	12.677	4.482
RA	93.690	25.730	9.725		RA	93.023	17.058	5.394
**Old females**	**Mean**	**SD**	**SE**		**Young females**	**Mean**	**SD**	**SE**
Veh	100.000	22.749	8.043		Veh	100.000	19.075	6.744
RA	97.934	17.120	6.053		RA	84.939	19.739	5.475

The final number of mice in the 24-month-old group was RA: males=7, females=7; Control: males=5, females=8; the final number of mice in the 3-month-old group was RA: males=10, females=13; Control: males=8, females=8 for HDL.

HDL (mMol/L)								
**Old males**	**Mean**	**SD**	**SE**		**Young males**	**Mean**	**SD**	**SE**
Veh	100.000	12.931	5.783		Veh	100.000	15.511	5.484
RA	89.599	31.381	11.861		RA	100.316	19.353	6.120
**Old females**	**Mean**	**SD**	**SE**		**Young females**	**Mean**	**SD**	**SE**
Veh	100.000	35.321	12.488		Veh	100.000	16.220	5.735
RA	99.846	19.119	6.760		RA	81.053	18.202	5.048

The final number of mice in the 24-month-old group was RA: males=7, females=6; Control: males=5, females=7; the final number of mice in the 3-month-old group was RA: males=10, females=13; Control: males=9, females=8 for LDL.

LDL (mMol/L)								
**Old males**	**Mean**	**SD**	**SE**		**Young males**	**Mean**	**SD**	**SE**
Veh	100.000	38.054	17.018		Veh	100.000	23.730	7.910
RA	80.643	38.087	14.395		RA	94.977	12.096	3.825
**Old females**	**Mean**	**SD**	**SE**		**Young females**	**Mean**	**SD**	**SE**
Veh	100.000	33.706	12.740		Veh	100.000	9.653	3.413
RA	83.712	57.457	23.457		RA	109.865	5.949	1.650

The final number of mice in the 24-month-old group was RA: males=7, females=7; Control: males=5, females=8; the final number of mice in the 3-month-old group was RA: males=10, females=12; Control: males=9, females=8 for FFA.

FFA (mMol/L)								
**Old males**	**Mean**	**SD**	**SE**		**Young males**	**Mean**	**SD**	**SE**
Veh	100.000	37.025	16.558		Veh	100.000	44.890	14.963
RA	107.366	53.883	20.366		RA	164.490	48.968	15.485
**Old females**	**Mean**	**SD**	**SE**		**Young females**	**Mean**	**SD**	**SE**
Veh	100.000	35.930	12.703		Veh	100.000	37.833	13.376
RA	150.287	73.089	25.841		RA	51.524	18.944	5.469

The final number of mice in the 24-month-old group was RA: males=7, females=7; Control: males=5, females=8; the final number of mice in the 3-month-old group was RA: males=10, females=12; Control: males=9, females=8 for TAG.

TRIGLYCERIDES (mMol/L)								
**Old males**	**Mean**	**SD**	**SE**		**Young males**	**Mean**	**SD**	**SE**
Veh	100.000	5.336	2.386		Veh	100.000	45.779	15.260
RA	117.460	29.554	11.170		RA	80.091	29.426	9.305
**Old females**	**Mean**	**SD**	**SE**		**Young females**	**Mean**	**SD**	**SE**
Veh	100.000	24.236	8.569		Veh	100.000	23.224	8.211
RA	124.351	22.868	8.643		RA	67.659	9.494	2.741

Supplementary figures

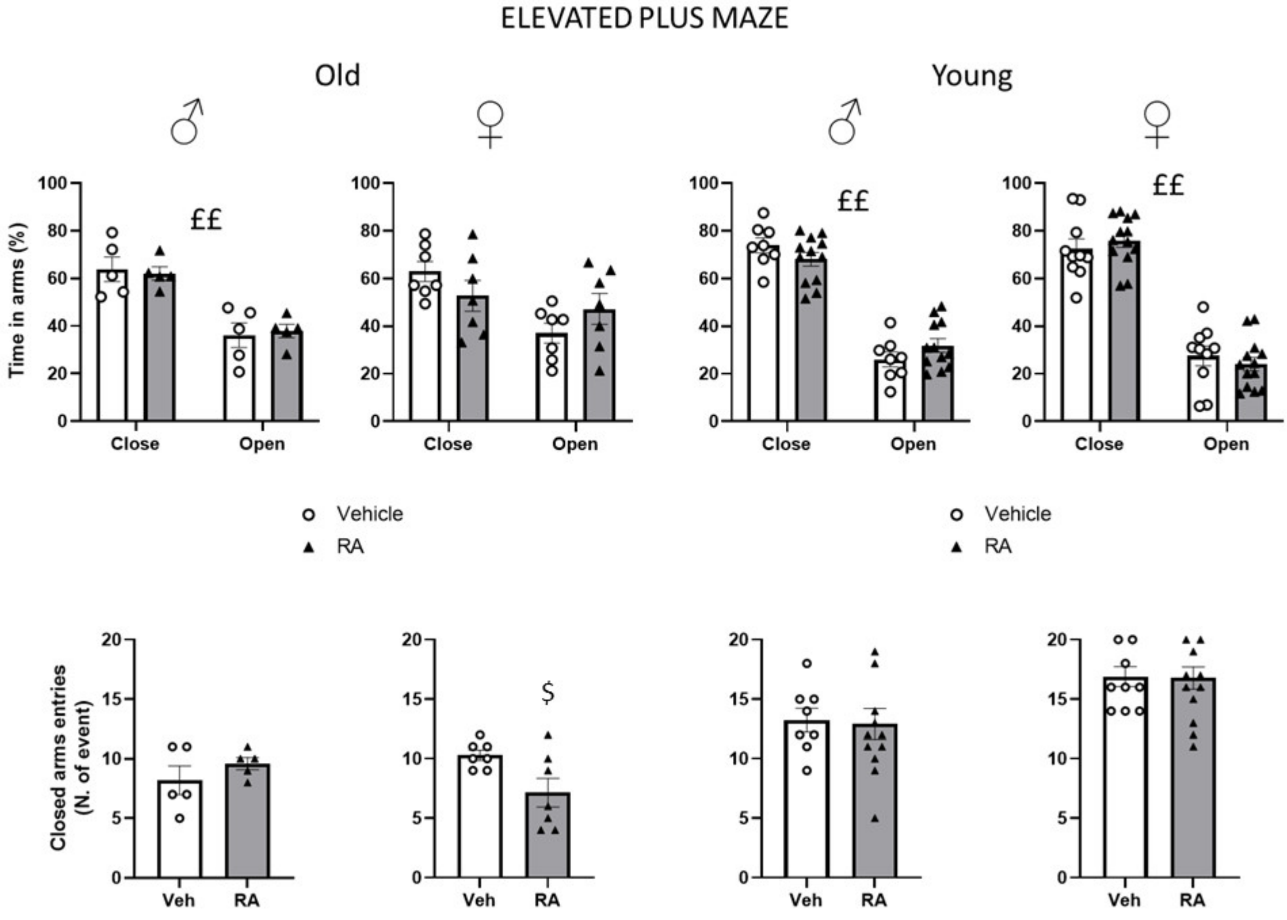

Figure S2. Elevated Plus Maze - Old males showed a preference for the closed arms of the maze while this was not observed in same age females. Only old RA-females showed a reduced frequency to visit the closed arms suggesting reduced anxiety. Both male and female young mice showed a strong preference for the closed arms and no difference was observed in closed arms entries. Data are expressed as mean ± SEM. White dots and black triangles represents individual observations for Veh and RA mice, respectively. ££ $p < 0.01$ main effect of repeated measures (zone: Close vs. Open); \$ $p < 0.05$ main effect of treatment; old: N=5-8/group; young: N=9-13/group.

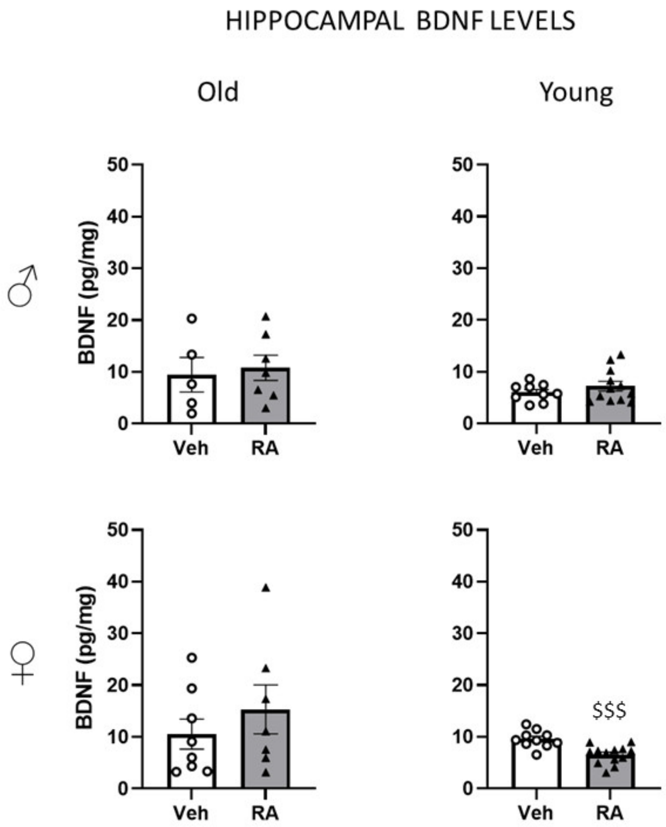

Figure S3. Hippocampal BDNF levels - RA significantly reduced hippocampal BDNF levels in young females. No difference was observed in young males as well as in old mice. Data are expressed as mean ± SEM. White dots and black triangles represents individual observations for Veh and RA mice, respectively. $$$ *p* <0.001 main effect of treatment; old: N=5-8/group; young: N=9-13/group.

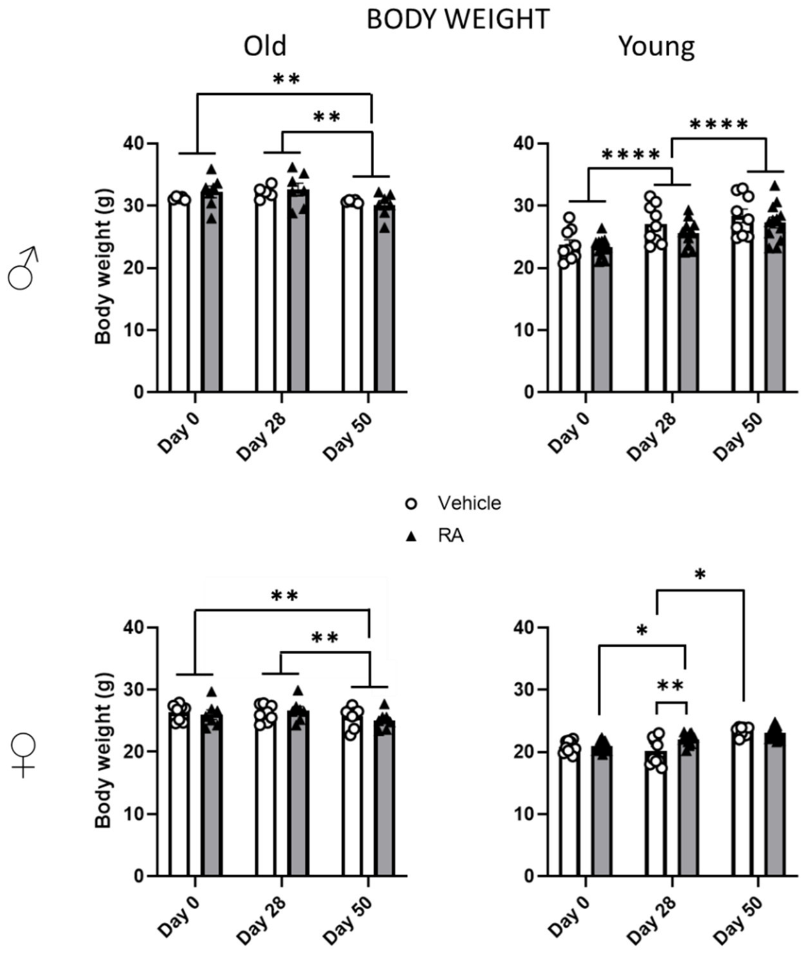

Figure S4. Body weight - Old subjects were overall characterised by a decrease in body weight at day 50. By contrast, young mice were characterised by an increase in body weight overtime regardless of treatment. As for females while RA-treated subjects showed an increase in body weight at day 28 in control females this increase was apparent at day 50. Data are expressed as mean ± SEM. White dots and black triangles represents individual observations for Veh and RA mice, respectively. Stars are used to indicate Tukey's post hoc comparisons: * $p < 0.05$, RA-day28 vs RA-day0 and Veh-day50 vs Veh-day28; ** $p < 0.01$, day50 vs day28 and day0 and RA-day28 vs Veh-day28, **** $p < 0.0001$, day28 vs day0 and day50 vs day28; old: N=5-8/group; young: N=9-13/group.

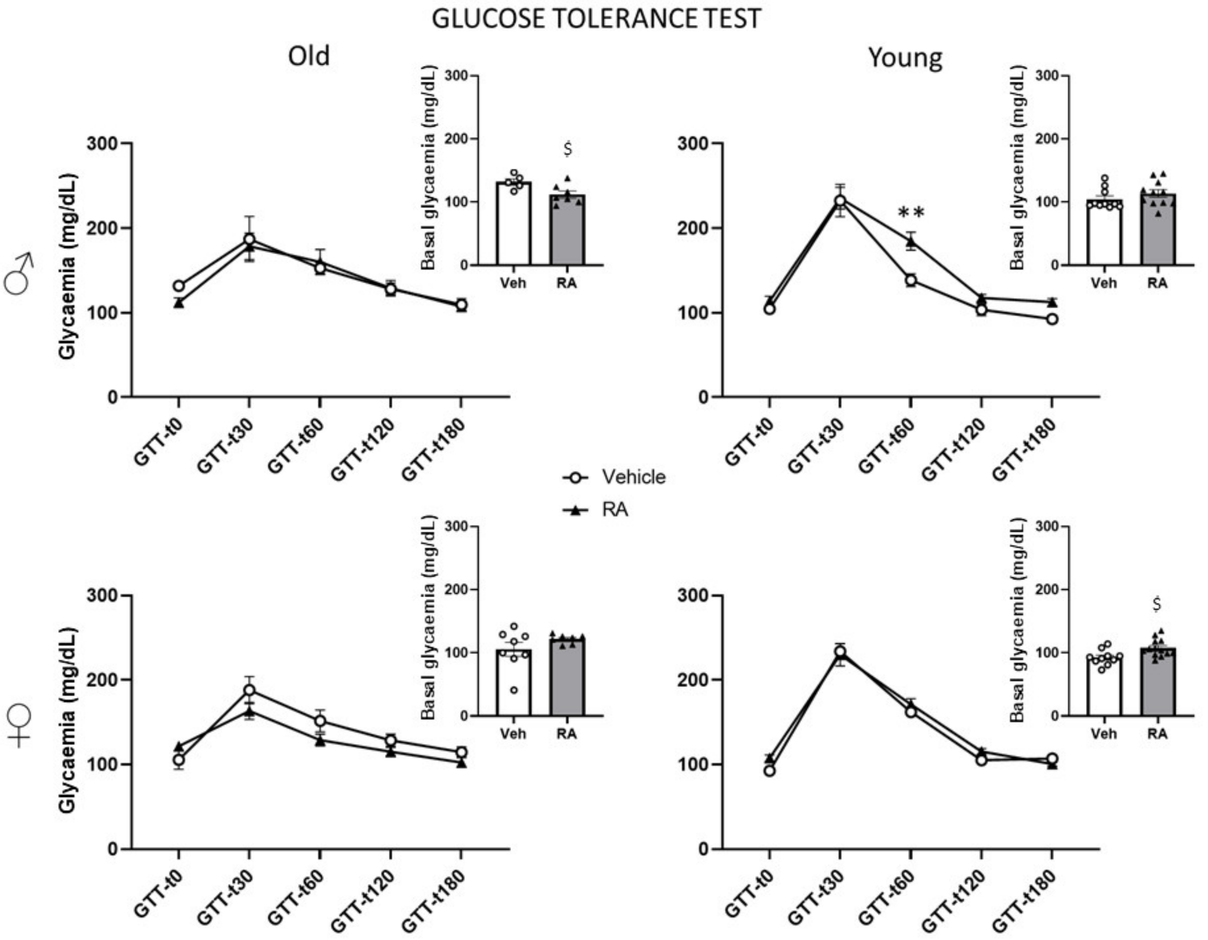

Figure S5. Glucose Tolerance Test - RA decreased the levels of basal glycaemia in old males and an overall slight trend in old RA females was also observed. By contrast, in young mice, RA increased glycaemia levels at T60 in males and at T0 in females. Data are expressed as mean ± SEM. White dots and black triangles represents individual observations for Veh and RA mice, respectively. $ *p* < 0.05 main effect of treatment; ** *p* < 0.01 Tukey's test RA-T60 vs Veh-T60; old: N=5-8/group; young: N=9-13/group.

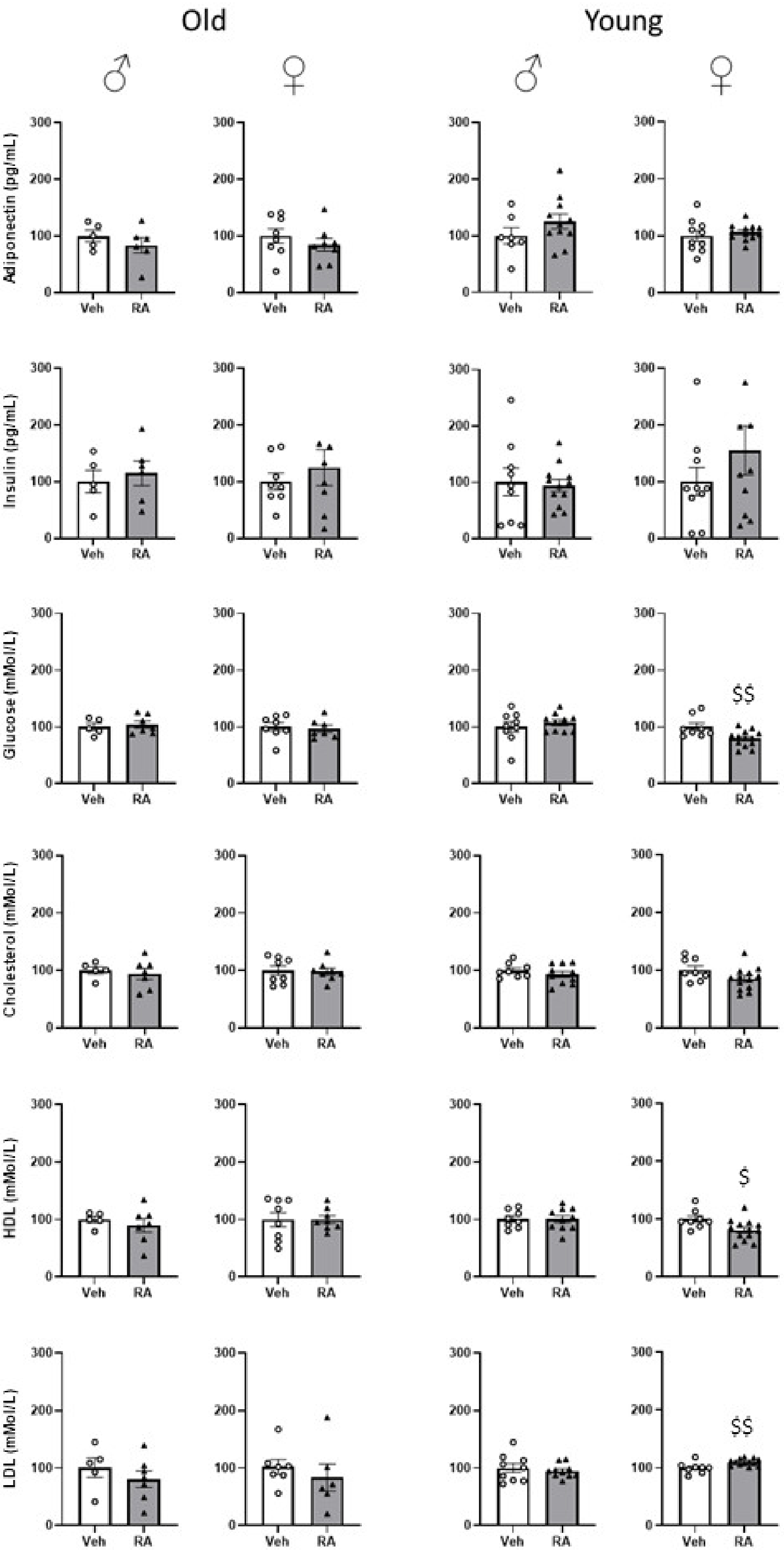

Figure S6. Serum and plasmatic parameters - No difference due to RA was observed in adiponectin and insulin serum levels in both old and young subjects. Moreover, RA treatment did not affect the lipid and glucose profile in old mice and young males while it decreased plasmatic glucose and HDL levels and increased LDL levels in young females. Data are expressed as mean ± SEM. White dots and black triangles represents individual observations for Veh and RA mice, respectively. \$ $p < 0.05$, \$\$ $p < 0.01$ main effect of treatment; old: N=5-8/group; young: N=9-13/group.
