# Supplementary material for: Rosmarinic Acid Improves Cognitive Abilities and Glucose Metabolism in Aged C57Bl/6N Mice While Disrupting Lipid Profile in Young Adults in a Sex-Dependent Fashion
Source: Nutrients. 2023 Jul 28;15(15):3366. doi: 10.3390/nu15153366 (PMC10421458; doi:10.3390/nu15153366)
Supplement: Supplementary file 1 [file nutrients-15-03366-s001.zip › suppl_Diet composition.pdf]

1320

Maintenance diet for rats and mice

The 1320 formula is a cereal-based (soy, wheat, corn) fixed formula which is free of alfalfa and fish/animal meal and deficient in nitrosamines. This maintenance diet was designed as complete feedingstuff for rats/mice from an age of 8 weeks.

Available as

1321 - Powder
1324 - 10mm pellets
1328 - Hybridpellet
1329 - Extrudate

Product variations

The 1320 formula is optionally also available in one or a combination of the following variants.

Variant P
deficient in phytoestrogens
Variant Fortified
autoclavable, increased vitamin content, coated on request
Variant Irradiated
irradiated with 5 to 50kGy
Variant TPF
total pathogen free

The contents may vary for these variants.

Metabolized energy

Content	Value		unit
Fat	367	(11%)	kcal/kg
Protein	768	(24%)	kcal/kg
Carbonhydrates	2,091	(65%)	kcal/kg

crude nutrients and moisture

Content	Value		unit
Moisture	113,426	(11.3%)	mg/kg
Crude Ash	58,553	(5.9%)	mg/kg
Crude Fibre	60,744	(6.1%)	mg/kg
Crude Fat	40,791	(4.1%)	mg/kg
Crude Protein	192,111	(19.2%)	mg/kg
Nitrogenfree extractives	534,374	(53.4%)	mg/kg

Altromin Spezialfutter GmbH & Co. KG
Im Seelenkamp 20, 32791 Lage, Germany
info@altromin.de - www.altromin.com

Carbonhydrates

Content	Value	unit
Monosaccharides	0	mg/kg
Disaccharides	47,779	mg/kg
Polysaccharides	391,216	mg/kg

Minerals

Content	Value	unit
Calcium	7,012	mg/kg
Potassium	8,797	mg/kg
Magnesium	2,220	mg/kg
Sodium	2,128	mg/kg
Phosphorus	5,014	mg/kg

Trace elements

Content	Value	unit
Aluminium	79.37	mg/kg
Chlorine	3,484.07	mg/kg
Iron	192.51	mg/kg
Flourine	2.80	mg/kg
Iodine	1.66	mg/kg
Cobalt	0.34	mg/kg
Copper	12.81	mg/kg
Manganese	95.06	mg/kg
Molybdenum	1.10	mg/kg
Sulfur	1,141.22	mg/kg
Selenium	0.25	mg/kg
Zinc	95.18	mg/kg

Altromin Spezialfutter GmbH & Co. KG
Im Seelenkamp 20, 32791 Lage, Germany
info@altromin.de - www.altromin.com

Added vitamins

Content	Standard	Fortified	unit
Vitamin A	15,000	26,250	IU/kg
Vitamin D3	600	1,050	IU/kg
Vitamin E	75	132	mg/kg
Vitamin K3	3	5	mg/kg
Vitamin B1	18	32	mg/kg
Vitamin B2	12	21	mg/kg
Vitamin B6	9	16	mg/kg
Vitamin B12	24	42	µg/kg
Nicotinic acid	36	63	mg/kg
Pantothenic acid	21	37	mg/kg
Folic acid	2	4	mg/kg
Biotin	167	205	µg/kg
Choline chloride	600	1,050	mg/kg
Vitamin C	36	63	mg/kg

Amino acids

Content	Value	unit
Alanine	8,292	mg/kg
Arginine	11,175	mg/kg
Aspartic acid	15,297	mg/kg
Cystine	3,182	mg/kg
Glutaminc acid	38,695	mg/kg
Glycine	8,028	mg/kg
Histidine	4,351	mg/kg
Isoleucine	7,408	mg/kg
Leucine	13,214	mg/kg
Lysine	8,101	mg/kg
Methionine	2,709	mg/kg
Phenylalanine	8,221	mg/kg
Proline	12,523	mg/kg
Serine	8,965	mg/kg
Threonine	6,399	mg/kg
Tryptophan	2,342	mg/kg
Tyrosine	5,849	mg/kg
Valine	8,634	mg/kg

Altromin Spezialfutter GmbH & Co. KG
Im Seelenkamp 20, 32791 Lage, Germany
info@altromin.de - www.altromin.com

Fatty acid

Content	Value	unit
Arachidic acid C-20:0	35	mg/kg
Eicosanoic acid C-20:1	44	mg/kg
Alpha-Linolenic acid C-18:3	2,210	mg/kg
Linolenic acid C-18:2	16,152	mg/kg
Palmitic acid C-16:0	3,878	mg/kg
Stearic acid C-18:0	1,187	mg/kg
Oleic acid C-18:1	6,823	mg/kg

Altromin Spezialfutter GmbH & Co. KG
Im Seelenkamp 20, 32791 Lage, Germany
info@altromin.de - www.altromin.com
